# A Retrospective Cohort Study of Acute Epiglottitis in Adults

**DOI:** 10.5811/westjem.2021.8.52657

**Published:** 2021-11-05

**Authors:** Patrick Felton, Lucienne Lutfy-Clayton, Liza Gonen Smith, Paul Visintainer, Niels K. Rathlev

**Affiliations:** * University of Massachusetts Medical School-Baystate, Department of Emergency Medicine, Springfield, Massachusetts; † University of Massachusetts Medical School-Baystate, Department of Epidemiology and Biostatistics, Springfield, Massachusetts

## Abstract

**Introduction:**

Adult epiglottitis is a disease process distinct from pediatric epiglottitis in microbiology, presentation, and clinical course. While traditionally considered more indolent and benign than in children, adult epiglottitis remains a cause of acute airway compromise with a mortality rate from 1–20%. Our objective was to characterize the disease course and evaluate the rate and type of airway management in this population at a tertiary, academic referral center.

**Methods:**

We conducted a retrospective chart review of all adult patients (age ≥ 18) who were definitively diagnosed with infectious “epiglottitis,” “supraglottitis,” or “epiglottic abscess” by direct or indirect laryngoscopy during a nine-year period. Double data abstraction and a standardized data collection form were used to assess patient demographic characteristics, presenting features, and clinical course. The primary outcome was airway intervention by intubation, cricothyroidotomy, or tracheostomy, and the secondary outcome was mortality related to the disease.

**Results:**

Seventy patients met inclusion criteria. The mean age was 50.2 years (standard deviation ± 16.7), 60% of the patients were male, and 14.3% were diabetic. Fifty percent had symptoms that were present for ≥ 48 hours; 38.6% had voice changes, 13.1% had stridor, 12.9% had fever, 45.7% had odynophagia, and 47.1% had dysphagia noted in the ED. Twelve patients (17.1%) received an acute airway intervention including three who underwent emergent cricothyroidotomy, and one who had a tracheostomy. Two patients died and one suffered anoxic brain injury related to complications following difficult airway management.

**Conclusion:**

In this case series the majority of patients (82.9%) did not require airway intervention, but a third of those requiring intervention (5.7% of total) had a surgical airway performed with two deaths and one anoxic brain injury. Clinicians must remain vigilant to identify signs of impending airway compromise in acute adult epiglottitis and be familiar with difficult and failed airway algorithms to prevent morbidity and mortality in these patients.

## INTRODUCTION

The incidence of epiglottitis in the pediatric population has fallen significantly since the widespread use of the *Haemophilus influenzae* type B (HIB) vaccine in the United States.^1^ Epiglottitis in the adult population remains a distinct process from pediatric disease with respect to microbiology, spectrum of presenting symptoms, and an often benign clinical course.^2^ However, adult epiglottitis remains a recognized cause of acute airway compromise with an associated mortality rate reported from 1–20%.^3^,^4^ A growing body of literature has demonstrated that the incidence of epiglottitis in the adult population is increasing in the post-HIB vaccine era.^1^,^5^

Symptoms of adult epiglottitis can include sore throat, fever, dysphagia, dyspnea, stridor, drooling, and acute respiratory compromise. Differentiating acute epiglottitis from other, more benign, causes of sore throat can be difficult and can lead to delays in diagnosis and subsequent increase in airway-related mortality.^6^ There is general agreement that a “selective” approach to airway management is appropriate with adult epiglottitis. Selecting which patients will benefit from airway intervention, however, remains challenging. Most patients appear to do well with conservative management with antibiotics and airway monitoring. Unfortunately, there appears to be a subset of patients without prominent respiratory symptoms initially who have rapid disease progression and acute airway compromise.^5^

Three cases of adult epiglottitis who presented to our emergency department (ED) over a two-year period requiring emergent surgical airway intervention prompted our review. We performed a retrospective study to evaluate the clinical presentation of acute epiglottitis in adults at our institution and to characterize the clinical course and need for airway intervention.

## BRIEF CASE REPORT

A 35-year-old male presented to the ED at 11 am with complaints of two days of sore throat accompanied by fevers and chills. He went to a clinic and was referred to the ED due to concern for peritonsillar abscess. Triage vital signs were as follows: temperature 102.7°F, pulse 110 beats per minute; 26 respirations per minute, oxygen saturation 98%, and blood pressure of 141/75 millimeters mercury. His body mass index was 46. The triage nurse commented that the patient was unable to speak due to pain but was in “no respiratory distress.” He was seen 30 minutes after arrival by a physician assistant who found the pharynx to be injected with exudates, but the posterior structures could not be assessed due to swelling and discomfort. No anterior neck swelling was noted, but this was also difficult to assess due to obesity.

Intravenous clindamycin and steroids were administered. The attending physician attempted to spray atomized lidocaine into the oral cavity to facilitate visual exam. The patient quickly developed laryngospasm and respiratory distress. At this point, the emergency physician used a nasopharyngoscope to identify swollen and bloody epiglottic and supraglottic structures. Anesthesia and trauma surgery attendings were paged overhead. While awaiting the anesthesia and surgical attendings, the patient developed stridor and diaphoresis. The emergency physician proceeded with ketamine dissociation and video-orotracheal laryngoscopy with a GlideScope (Verathon Inc, Bothell, WA). The vocal cords were visualized, but the operator was unable to pass an endotracheal tube (ETT). Ventricular-fibrillation cardiac arrest followed.

The emergency physician attempted a cricothyroidotomy, but he was unable to pass an ETT. A surgical resident unsuccessfully attempted a surgical airway. Anesthesiology unsuccessfully simultaneously attempted oropharyngeal intubation. The trauma surgery attending had difficulty identifying anatomical landmarks but was able to perform a cricothyroidotomy and secure a 6-0 ETT in the trachea. The patient regained spontaneous circulation shortly after the surgical airway was secured following approximately 12 minutes of chest compressions and defibrillation. The patient remained in the intensive care unit for two days with some spontaneous movements and respirations, receiving antibiotics and supportive care. Magnetic resonance imaging on day 2 after the arrest revealed anoxic encephalopathy. The patient was made comfort measures only (CMO) and expired.

**Population Health Research Capsule**

What do we already know about this issue?

*Epiglottitis can lead to acute loss of airway patency in adults.*

What was the research question?

*Are there factors that can predict the need for advanced airway intervention in adults presenting with epiglottitis?*

What was the major finding of the study?

*Stridor, dyspnea, and voice changes predict need for advanced airway in adult epiglottitis.*

How does this improve population health?

*Avoiding intubation in patients without high-risk features can reduce healthcare costs and preserve ICU beds. Early recognition of epiglottitis can reduce mortality.*

## METHODS

### Study Design

Baystate Medical Center is a 716-bed tertiary academic referral and Level I trauma center and the Western regional campus of the University of Massachusetts Medical School. The ED has an annual census of over 90,000 adult patients. The electronic health records (EHR) of all adult patients (age 18 and older) treated in our adult ED between January 2009–December 2017 who met inclusion criteria underwent retrospective chart review. Approval was obtained from the Baystate Medical Center Investigational Review Board prior to the start of the retrospective chart review.

### Selection of Participants

Patients were selected based on a diagnosis of “epiglottitis,” “epiglottic abscess,” or “supraglottitis” by *International Classification of Diseases, 9^th^ Revision* codes (464.5, 464.51, J04.30, 464.3, 464.31, J05.10). The diagnosis of epiglottitis was established in all cases by direct or indirect laryngoscopy findings. We excluded patients if they were less than 18 years, did not have a final diagnosis of “epiglottitis,” “epiglottic abscess,” or “supraglottitis,” or had chronic tracheostomies. We reviewed a total of 122 charts, and 52 were excluded due to either an obvious miscode, duplication related to interfacility transfer, or not meeting inclusion criteria.

### Intervention

After review, 70 patients met the inclusion criteria and their ED and EHRs were retrospectively reviewed by three emergency medicine resident physicians using double data abstraction and a standardized data collection form (Appendix 1). We assessed patient demographics, presenting symptoms, physical and radiographic findings, laboratory data, treatment, clinical course, complications, and final outcome. All patient information was saved on a secure Research Electronic Date Capture (REDCap, Vanderbilt University, TN) software platform.

### Measurements

Prior to the review of these charts the data abstraction form was reviewed by the investigators and chart reviewers to establish consensus on patient and clinically significant variables to be included. The three chart reviewers subsequently independently reviewed the EHRs and abstracted the data based on the agreed-upon variables. A fourth reviewer reviewed all cases, and inter-rater reliability was calculated between the first three reviewers in aggregate and the fourth.

### Outcomes

Using retrospective chart review of the clinical presentation of adult epiglottitis and the clinical course, we calculated the number and percentage of patients requiring airway intervention, the primary outcome. Clinical predictors of the necessity for airway intervention were analyzed. Secondary outcomes included the methods used for instituting a definitive airway and incidence of anoxic brain injury and death.

### Analysis

For descriptive statistics, means and standard deviations are presented for continuous measurements, and proportions are presented for categorical variables. For comparisons between patients classified by whether they received airway management, we used t-tests for continuous variables and Fisher’s exact test for categorical variables. Significance testing was conducted at a critical test level of 5%.

## RESULTS

We identified 70 cases of adult epiglottitis that met inclusion criteria during the study period. Demographic data and significant comorbidities are listed in Table 1. There were 28 females and 42 males. Patients ranged from 19–96 years of age, with a mean of 50.2 years. Ten (14.3%) patients had a documented history of diabetes, three (4.3%) had a documented history of human immunodeficiency virus, three (4.3%) had a history of chronic inflammatory disease requiring steroids (inflammatory bowel disease, inflammatory arthropathy), and seven patients (10%) had a history of alcohol abuse documented. A total of 44 (62.9%) patients had a recorded race of “White,” four (5.7%) patients were Black, one patient was Asian, and one was Native American. Race was not listed or could not be determined from the EHR for the remaining 20 (28.6%) patients.

We abstracted clinical characteristics and initial interventions and summarized them in Table 2. The most common presenting symptoms were dysphagia and odynophagia, which were reported by 33 (47.1%) and 32 (45.7%) patients, respectively. Sore throat was reported by 36 patients (51.4%). Nine patients (12.9%) had a recorded fever on initial presentation to the ED, and 11 patients (15.9%) developed a fever later during their hospitalization. Two patients reported a symptom duration of less than 12 hours, 12 reported a symptom duration of between 13–24 hours, and 33 patients (50.0%) reported a symptom duration of greater than 49 hours. Upon initial presentation, eight (13.1%) patients had stridor and five (9.1%) had trismus. Sixty-three patients (90.0%) received corticosteroids. Twenty-three patients (32.9%) were admitted to an intensive care unit bed; 16 patients (22.9%) were admitted to an intermediate care/stepdown bed, and 26 patients (37.1%) were admitted to a medical floor bed.

Results of diagnostic test are summarized in Table 3, Two patients had positive blood cultures documented in the EHR. Thirty patients had confirmed negative blood cultures, and the remainder did not have blood cultures performed or the results were unavailable. Rapid antigen test for Group A streptococcus was positive for two patients and negative for 25. Forty-one patients (59.4%) had a computed tomography (CT) of the neck suggestive of epiglottitis. Three patients had a CT that did not demonstrate epiglottitis.

There were three deaths in the study population. Two patients died during the acute phase of their presentation. One patient was found to have hypoxic encephalopathy as a result of difficult airway management and was made CMO several days later. The remaining 67 patients were ultimately discharged neurologically intact.

Eight patients (11.4%) underwent intubation without requiring a surgical airway. Three patients had an emergency cricothyroidotomy performed (4.3%). One patient underwent emergent tracheostomy (1.4%). Fifty-eight patients (82.9%) did not require advanced airway management. The eight patients who underwent intubation without requiring a surgical airway were intubated using a fiberoptic device. Seven of those fiberoptic intubations were performed by an anesthesiologist, and one was intubated by an otolaryngologist. The ETT sizes used are listed in Table 4. There were no patient demographic factors associated with need for advanced airway management. See Table 5 for airway management by patient characteristics. Historical factors associated with need for airway management include the presence of stridor, dyspnea, and voice alteration by univariate analysis (Table 6).

Our inter-rater reliability was 91% for the question “Should this patient be included?” and 98.5% for the question “Did this patient have advanced airway management?” These were calculated as simple percentages: the number of findings in agreement over the total number of cases.

## DISCUSSION

Epiglottitis has long been recognized as a severe disease with potential for airway catastrophe. Allen et al found that mortality from acute epiglottitis decreased after widespread adoption of HIB vaccination and that adults in the US are now more likely than children to die of acute epiglottitis.^7^ Our series found statistically significant correlations between the presence of dyspnea, stridor, and voice changes, and the likelihood of airway intervention. While most patients with dyspnea and/or voice changes will have neither epiglottitis nor require intubation, the majority of patients experiencing stridor are at risk of acute airway compromise and in our cohort heralded both the need for airway intervention and for surgical airway management at a much higher incidence than those without stridor. Airway interventions were undertaken in 17% of the patients with epiglottitis within our cohort. This is slightly higher than the 10.9% rate found in a recent meta-analysis of predictors of airway intervention in adult epiglottitis.^8^ Our case series does highlight some important points to consider when assessing patients with epiglottitis.

### Timing of Airway Intervention

When evaluating epiglottitis and the institutional resources needed for close observation, it may be helpful to note that, in our case series, no patients required airway intervention after 12 hours of observation. While overall time course and the rate of change of symptoms should be considered, the varying time course of progression can make determining the appropriate length of observation difficult. Given that no patients in our cohort decompensated more than 12 hours after arrival, observation in the highest level of care available with an available surgeon for that period of time may be a reasonable approach.

### Location of airway intervention

The location most appropriate for airway intervention in epiglottitis patients has traditionally been the operating room (OR). In this series 55% of the airway interventions took place in the ED, including one in a community affiliate ED, and 45% took place in the OR, including one in a community affiliate. Among these airways, only one resulted in an anoxic brain injury. In this case, the airway compromise immediately followed an attempt at visualizing the posterior pharynx with the aid of atomized lidocaine. This patient was referred to the ED due to concern for peritonsillar abscess, and the documented examination followed accepted practice with this presentation. One emergency medicine textbook suggests that use of atomized lidocaine is appropriate and recommends evaluation of the airway in suspected epiglottitis.^9^ Balancing prudence with resource utilization continues to be a nuanced element of emergency medicine practice.

### Method of airway intervention

Non-surgical airways were successful in 72% of the patients requiring an airway intervention within our cohort. These airways were performed by anesthesiologists, otolaryngologists and emergency clinicians and included awake, rapid sequence intubation, nasotracheal and orotracheal approaches with video-assisted laryngoscopy, direct laryngoscopy, and fiberoptic devices. Surgical airways, including both emergent cricothyrotomies and tracheostomies, accounted for the remainder of the airways in our cohort and were ultimately successful. While the literature traditionally supported universal surgical intervention without attempt at orotracheal or nasotracheal intubation, this recommendation has been replaced with awake fiberoptic intubation approaches over the past 20 years.^10^, ^11^ In cases of presumed or known epiglottitis, early consultation with intensivists, anesthesiologists, otolaryngologists, and surgeons should be strongly considered to develop an airway plan that can be quickly implemented if decompensation occurs.

In contrast to pediatric patients, adults with epiglottitis who are not in extremis may benefit from close monitoring, and antibiotics and steroids, although prophylactic intubation can also be considered. Additionally, in contrast to treatment of pediatric disease, this more conservative approach may be considered in adults given the larger diameter of the adult larynx.^12^ Intravenous fluids and humidified oxygen may help to limit the risk of sudden airway obstruction and should be considered by clinicians in the process of evaluating a patient with suspected epiglottitis.^13^ Administration of humidified oxygen was not documented in the cases we reviewed. Clinical judgment should always be paramount when considering location and method of airway intervention for adults with epiglottitis.

### Imaging vs Inspection

**Figure 1:**
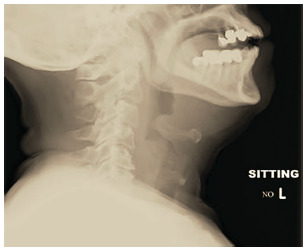
Note the pronounced swelling of the epiglottis, often referred to as “thumbprinting.”

Imaging for clinically stable patients with possible epiglottitis can be considered, although direct visualization of the epiglottis remains the gold standard for diagnosis. The overwhelming majority of our patients received a preliminary diagnosis of epiglottitis based on imaging with CT, which remains an appropriate modality for many reasons, including expediency, accuracy at diagnosing a wide variety of pathology, and widespread availability. A notable downside of the use of CT is the need for patients to be in the supine position. A trial of supine positioning in the stable patient should be performed in the department with the physician at bedside prior to CT. Lateral neck radiograph can performed with the patient upright with neck extended, and the emergency physician should be familiar with the appearance of pathognomonic “thumbprinting” (Figure 1).

Other findings visible on radiography include thickening of the aryepiglottic folds, prevertebral soft tissue swelling, and expansion of the hypopharynx. Sensitivity and specificity of plain radiography varies from 38–98%,^4^, ^14^ and may be useful in institutions without access to CT or fiberoptic nasopharyngoscopy capability. Looking forward, point-of-care ultrasound may represent a safer alternative for identification of epiglottitis, as it can be performed at bedside in the patient’s position of comfort with limited aggravation.^15^ With all these modalities, care should be taken to perform them only on patients who are not in extremis.

## LIMITATIONS

Our methodology involved chart abstraction of variables regarding time course, subjective symptoms, and specific physical exam findings. As with most retrospective chart reviews, these variables were not universally present in the documentation. This variability is likely related to patient discomfort and the critical nature of these presentations, limiting history and real-time documentation in favor of marshaling resources. The paucity of data within charts, especially those that were handwritten, may cause significant associations to have been overlooked. Our results should be considered hypothesis-generating and require prospective analysis in a multicenter trial for confirmation

Several patients who were initially included had complex head and neck cancers, supraglottic infections related to pre-existing tracheostomies, and other pathology such as infectious mononucleosis and vasculitis. These cases were omitted after the research team decided that these patients were not in keeping with the objective of describing acute bacterial epiglottitis in patients with normal anatomy. As only 12 patients required an advanced airway, only univariate analysis was performed. Our attempts at logistic regression to adjust for confounding factors led to very wide confidence intervals that did not represent any meaningful data associations. Thus, our review is hypothesis-generating rather than hypothesis-testing.

In concert with the Gilbert and Lowenstein^16^ recommendations for chart reviews, we followed most of the principal strategies. Opportunities for better adherence include providing chart abstractors with “practice” medical records as part of training in the use of RedCAP database; while the abstraction form was standardized and uniform, multiple types of emergent surgical airways were not anticipated. Formal review of coding rules did not occur at reviewer meetings, and blinding reviewers to the research outcomes was not practical given the complexity of data being extracted from narrative reports. Lastly, the abstraction form failed to clearly define the history of tobacco use and “chronic medical conditions.”

## CONCLUSION

Epiglottitis is a life-threatening diagnosis that, after HIB vaccination implementation, is now more deadly in adults at 0.015 per 100,000 than in pediatrics at 0.006 per 100,000.^7^ This case series found that the majority of adult patients diagnosed with epiglottitis in our system (82.9%) did not require airway intervention, but a third (5.7% of total) of those who did require intervention had a surgical airway and three deaths ultimately occurred. Clinicians must remain vigilant to identify signs of impending airway compromise in acute adult epiglottitis including dyspnea, voice change, and stridor. Emergency clinicians should be familiar with difficult and failed airway algorithms to prevent morbidity and mortality in these patients. Coordinating definitive airway management in conjunction with anesthesiologists, otolaryngologists, and surgeons is likely to offer the best chance for a successful outcome. For those patients presenting without clear indications for airway management, we suggest that clinicians consider close observation in the highest level of care for at least 12 hours to monitor for acute airway compromise.

## Supplementary Information







## Figures and Tables

**Table 1:** Demographic data and comorbidities.

Demographic Data and Comorbidities	N (%) ie, Number of Cases and Percent of Total	
Age		
Mean age	50.2	
Age range	19–96	
Gender		
Female	28	40.0%
Male	42	60.0%
Comorbidities		
Diabetes	10	14.3%
HIV	3	4.3%
Chronic inflammation	3	4.3%
Alcohol abuse	7	10.0%
Race		
White	44	62.9%
Black	4	5.7%
Asian	1	1.4%
Native American	1	1.4%
Other/Unknown	20	28.6%
Ethnicity		
Hispanic	22	31.9%

*HIV*, human immunodeficiency virus.

**Table 2:** Clinical characteristics and interventions.

Clinical Characteristics and Interventions	N(%) i.e. Number of Cases and Percent of Total	
Steroids		
Prednisone	3	4.3%
Dexamethasone	48	68.6%
Methylprednisolone	11	15.7%
Other	1	1.4%
Disposition		
ICU	23	32.9%
Intermediate	16	22.9%
Telemetry	1	1.4%
Floor	26	37.1
Home from ED	4	5.7%
Symptoms		
Dyspnea	7	10.0%
Dysphagia	33	47.1%
Odynophagia	32	45.7%
Drooling	12	17.1%
Voice change	27	38.6%
Sore throat	27	51.4%
Signs		
Stridor noted	8	13.1%
Trismus noted	5	9.1%
Fever on presentation	9	12.9%
Fever during hospitalization	11	15.9%
Time of onset		
< 12 hours	2	3.0%
12–24 hours	12	18.2%
25–48 hours	19	28.8%
> 49 hours	33	50.0%

*ICU*, intensive care unit; *ED*, emergency department.

**Table 3:** Diagnostic testing.

Diagnostic Testing	N(%) i.e. Number of Cases and Percent of Total	
Rapid strep testing		
Positive	2	2.9%
Negative	25	35.7%
Not performed or not recorded	43	61.4%
Radiology		
CT findings suggestive of epiglottitis	41	59.4%
CT negative	3	4.3%
Unknown whether CT was performed	2	2.9%
No CT performed	23	33.3%

*CT*, computed tomography.

**Table 4:** Airway interventions.

Airway Interventions	N(%) i.e. Number of Cases and Percent of Total	
Airway type		
Nonsurgical intubation	8	11.4%
Cricothyroidotomy	3	4.3%
Tracheostomy	1	1.4%
No advanced airway	58	82.9%
Method of Visualization		
Video	3	5.7%
Direct	4	7.5%
Fiberoptic nasopharyngoscopy	30	56.6%
Fiberoptic laryngoscopy	16	30.2%
Who performed direct visualization		
Emergency physician	3	5.7%
ENT physician	45	84.9%
Anesthesia	5	9.4%
Endotracheal tube size (nonsurgical)		
6.0	2	4.3%
6.5	1	1.4%
7.0	4	5.7%
8.0	1	1.4%
Endotracheal tube/tracheostomy size (surgical)		
6.0	2	4.3%
6.5	1	1.4%
7.0	1	1.4%

*ENT*, ear, nose, and throat.

**Table 5:** Airway management by patient characteristics.

Characteristic	No airway management (n = 58)	Advanced airway management (n = 12)	p-value
Age, mean (SD)	50.5 (17.9)	48.8 (10.1)	0.751
Male	35 (60.3%)	7 (58.3%)	1.000
Race			0.642
White	35 (60.3%)	9 (75.0%)	
Black	3 (5.2%)	1 (8.3%)	
Asian	1 (1.7%)	0 (0.0%)	
Native American	1 (1.7%)	0 (0.0%)	
Other/unknown	18 (31.0%)	2 (16.7%)	
Presence of diabetes	11 (15.7%)	4 (23.5%)	0.678
Presence of HIV	2 (3.4%)	1 (8.3%)	0.44
Chronic inflammatory disease on steroids	3 (5.2%)	0 (0.0%)	1.000
History of alcohol abuse	4 (6.9%)	3 (25.0%)	0.092
Stridor noted in the ED?	1 (2.0%)	7 (70.0%)	<0.001
Voice alteration	18 (31.0%)	8 (75.0%)	0.008
Dyspnea	2 (3.4%)	5 (41.7%)	0.001
Dysphagia	28 (48.3%)	5 (41.7%)	0.758
Odynophagia	28 (48.3%)	4 (33.3%)	0.526
Drooling	10 (17.2%)	2 (16.7%)	1.000
Sore throat	31 (53.4%)	5 (41.7%)	0.535
Fever at presentation	8 (13.8%)	1 (8.3%)	1.000
Fever during hospitalization	8 (13.8%)	3 (27.3%)	0.364

*SD*, standard deviation; *HIV*, human immunodeficiency virus; *ED*, emergency department.

**Table 6:** Airway management by patient presentation.

Patient characteristics	No airway management (n = 58)	Advanced airway management (n = 12)	P-value
Were there findings of epiglottitis on radiograph?			0.070
Yes	20 (34.5%)	2 (16.7%)	
No	3 (5.2%)	3 (25.0%)	
Unknown	1 (1.7%)	1 (8.3%)	
Imaging not done	34 (58.6%)	6 (50.0%)	
Radiographic findings of epiglottitis on CT?			0.436
Yes	24 (61.4%)	6 (50.0%)	
No	3 (5.3%)	0 (0.0%)	
Unknown	1 (1.8%)	1 (8.3%)	
Imaging not done	18 (31.6%)	5 (41.7%)	
Already on antibiotics?	16 (28.6%)	5 (45.5%)	0.301
Time since symptom onset (in hours)?			1.000
< 12 hours	2 (3.6%)	0 (0.0%)	
13–24 hours	10 (18.2%)	2 (18.2%)	
25–48 hours	16 (28.1%)	3 (27.3%)	
> 49 hours	27 (49.1%)	6 (54.5%)	
Who provided direct visualization?			0.001
Emergency physician	1 (2.3%)	2 (22.2%)	
ENT physician	42 (95.5%)	3 (33.3%)	
Anesthesiologist	1 (2.3%)	4 (44.4%)	
Location where visualization performed?			0.003
ED	21 (47.7%)	6 (66.7%)	
OR	2 (4.5%)	3 (33.3%)	
Inpatient care unit	21 (47.7%)	0 (0.0%)	
Trismus noted in the ED?	4 (8.5%)	1 (12.5%)	0.559
Tool used for visualization			0.324
Direct laryngoscopy	2 (4.5%)	2 (22.2%)	
Video-assisted laryngoscopy	3 (6.8%)	0 (0.0%)	
Fiberoptic nasopharyngoscopy	25 (56.8%)	5 (55.6%)	
Fiberoptic laryngoscopy	14 (31.8%)	2 (22.2%)	

*CT*, computed tomography.

*ENT*, ear, nose, and throat; *ED*, emergency department; *OR*, operating room.
