# Supplementary material for: A Retrospective Cohort Study of Acute Epiglottitis in Adults
Source: West J Emerg Med. 2021 Nov 5;22(6):1326–34. doi: 10.5811/westjem.2021.8.52657 (PMC8597686; doi:10.5811/westjem.2021.8.52657)
Supplement: Supplementary file 1 [file wjem-22-1326-s001.pdf]

Epiglottitis Data Abstraction Form

Please complete the survey below.

Thank you!

Patient MRN:	__________________
Age:	__________________
Gender:	◯ Female<br>◯ Male<br>◯ Other
Race:	◯ White<br>◯ Black<br>◯ Asian<br>◯ Native Hawaiian or Other Pacific Islander<br>◯ American Indian or Alaska Native<br>◯ Middle Eastern<br>◯ Other/Not Specified
Ethnicity	◯ Non-Hispanic<br>◯ Hispanic

Presenting Chief Complaint

What is the patient's stated chief complaint?	__________________
Associated Symptoms Noted in ED:	☐ Dyspnea<br>☐ Dysphagia<br>☐ Odynophagia<br>☐ Drooling<br>☐ Altered Voice<br>☐ ST<br>☐ Fever
Already on antibiotics?	◯ Yes<br>◯ No<br>◯ Unknown
Time since symptom onset (in hours)?	◯ < 12 hours<br>◯ 13-24 hours<br>◯ 25-48 hours<br>◯ > 49 hours<br>◯ Unknown
Have they seen a previous healthcare provider for these symptoms?	◯ Yes<br>◯ No<br>◯ Unknown
First Recorded Systolic Blood Pressure:	__________________

Was there any recorded SBP	◯ Yes<br>◯ No
First Recorded Temperature:	______________________
Was there any recorded temperature >/= 100.4?	◯ Yes<br>◯ No
Did the patient have stridor noted in the ED?	◯ Yes<br>◯ No<br>◯ Unknown
Did the patient have trismus noted in the ED?	◯ Yes<br>◯ No<br>◯ Unknown
Check any/all comorbidities suppressing immunity:	☐ None<br>☐ Diabetes<br>☐ HIV/AIDS<br>☐ Autoimmune Disease on Immunosuppression (RA, Crohn's, transplant patients, etc.)<br>☐ Inflammatory Conditions on Chronic Steroids (COPD, PMR, TA, etc.)<br>☐ Alcohol Abuse<br>☐ Other
Other Co-morbidity:	______________________

Labs and Imaging

WBC Count:	______________________
Blood Culture Results (if available):	◯ Positive<br>◯ Negative<br>◯ Not Available/Unknown
Positive for what organism?	______________________
Throat Culture Results (if available):	◯ Strep, Positive<br>◯ Strep, Negative<br>◯ Not Available/Unknown
Were there radiographic findings of epiglottitis on x-ray?	◯ Yes<br>◯ No<br>◯ Unknown<br>◯ Imaging Not Done
Were there radiographic findings of epiglottitis on CT scan?	◯ Yes<br>◯ No<br>◯ Unknown<br>◯ Imaging Not Done

Was there direct visualization?	◯ Yes<br>◯ No<br>◯ Unknown
How was there direct visualization?	◯ Direct Laryngoscopy<br>◯ Video Assisted Laryngoscopy<br>◯ Fiberoptic Nasopharyngoscopy<br>◯ Fiberoptic Laryngoscopy<br>◯ Other
If other, please specify:	__________________________________
Who provided the direct visualization?	◯ EM physician<br>◯ ENT physician<br>◯ Anesthesiologist<br>◯ Other
If other, please specify:	__________________________________
Where was there direct visualization?	◯ ED<br>◯ OR<br>◯ Other
If other, please specify:	__________________________________
What were the findings of the direct visualization?	◯ Abscess<br>◯ No Abscess<br>◯ Unknown

Management

Supplemental O2:	◯ Yes<br>◯ No<br>◯ Unknown<br>◯ No Airway Management
Airway Type:	◯ Yes<br>◯ No<br>◯ Unknown<br>◯ Cric<br>◯ Trach<br>◯ No Airway Management
ETT Tube Size:	◯ 6.0<br>◯ 6.5<br>◯ 7.0<br>◯ 7.5<br>◯ 8.0<br>◯ No Airway Management
ETT:	◯ Video<br>◯ Direct<br>◯ Fiberoptic<br>◯ No Airway Management

Antibiotic Choice:	◯ Unasyn<br>◯ Clindamycin<br>◯ Piperacillin/Tazobactam<br>◯ Levoflaxcin<br>◯ Other
What, if any, steroids were administered?	☐ None<br>☐ Prednisone<br>☐ Solumedrol<br>☐ Dexamethasone<br>☐ Other
Admit to:	◯ ICU<br>◯ Intermediate Care<br>◯ Telemetry<br>◯ Floor<br>◯ Expired

Outcome

Final Outcome (Check all that Apply):	☐ Death<br>☐ Anoxic Encephalopathy<br>☐ Discharged Home
Chronic Trach:	◯ Yes<br>◯ No<br>◯ Unknown
Total days in the ICU:	____________________
Total days in the hospital:	____________________
Initials of Staff Entering Data:	____________________
