# Supplementary material for: A Retrospective Cohort Study of Acute Epiglottitis in Adults
Source: West J Emerg Med. 2021 Nov 5;22(6):1326–34. doi: 10.5811/westjem.2021.8.52657 (PMC8597686; doi:10.5811/westjem.2021.8.52657)
Supplement: Supplementary file 2 [file wjem-22-1326-s002.docx]

**Appendix 2** Additional Tables

**Table 3a – Diagnostic Testing**

| X-ray findings suggestive of epiglottitis | 22 | 31.4% |
| --- | --- | --- |
| No x-ray findings | 6 | 8.6% |
| No x-ray done | 40 | 57.1% |
| Unknown | 2 | 2.9% |

| Blood cultures |  | |
| --- | --- | --- |
| Positive | 2 | 2.9% |
| Negative | 30 | 42.9% |
| Unavailable | 38 | 54.3% |

**Table 4a – Antibiotic Administration**

| **Were antibiotics administered prior to arrival at tertiary care center?** |  | |
| --- | --- | --- |
| No | 46 | 68.7% |
| Yes | 21 | 31.3% |

**Table 6a – Airway management by demographic factors**

| **Patient characteristics** | **No airway management**  **(n = 58)** | **Advanced airway management**  **(n = 12)** | **p-value** |
| --- | --- | --- | --- |

| Blood Culture Results |  |  | 0.001 |
| --- | --- | --- | --- |
| Positive | 1 (1.7%) | 1 (8.3%) |  |
| Negative | 20 (34.5%) | 10 (83.3%) |  |
| Not Available/Unknown | 37 (63.8%) | 1 (8.3%) |  |
| Throat Culture Results |  |  | 0.113 |
| Strep, Positive | 1 (1.7%) | 1 (8.3%) |  |
| Strep, Negative | 23 (39.7%) | 2 (16.7%) |  |
| Not Available/Unknown | 34 (58.6%) | 9 (75.0%) |  |

| Number of medical comorbidities |  |  | 0.034 |
| --- | --- | --- | --- |
| 0 | 37 (63.8%) | 3 (25.0%) |  |
| 1 | 15 (25.9%) | 7 (58.3%) |  |
| 2 | 6 (10.3%) | 2 (16.7%) |  |
